# Holmium laser enucleation of the prostate after prostatic urethral lift: the state of bother

**DOI:** 10.1007/s11255-026-05150-z

**Published:** 2026-05-04

**Authors:** Amir Patel, Jenny N Guo, Perry Xu, Alyssa McDonald, Allaa Fadl-Alla, Amy Krambeck

**Affiliations:** 1 Department of Urology, Oregon Health and Science University, Portland, OR USA; 2 Department of Urology, Northwestern University Feinberg School of Medicine, 676 N St. Clair, Suite 2300, Chicago, IL 60611 USA

**Keywords:** Benign prostatic hyperplasia, HoLEP, Prostatic urethral lift, Urolift

## Abstract

**Introduction:**

We sought to investigate the effect PUL has on pre-operative irritative voiding symptoms in patients presenting for HoLEP, and if this leads to different post-operative outcomes.

**Methods:**

This is a single-institution retrospective analysis of our prospective HoLEP registry. Propensity score matching of prostate size and history of urinary retention was used to match the patients who had prior PUL with patients without prior benign prostatic hyperplasia (BPH) surgery 1:2. We analyzed baseline demographics, operative details, post-operative outcomes, and pre-operative/post-operative International Prostate Symptom Score (IPSS) and Michigan Incontinence Symptom Index (MISI).

**Results:**

Of the 2114 patients who underwent HoLEP, 1242 had sufficient data. There were 59 patients who had a HoLEP after prior PUL and a control cohort was made consisting of 118 patients. At baseline, the control group had more alpha blocker use (69% vs 54% (*p*=0.05)) and higher American Association of Anesthesia scores. Enucleation efficiency was similar between groups (1.82 vs 1.94 g/min (*p*=0.634)) but morcellation efficiency was lower in the PUL arm (9.8 vs 7.2 g/min (*p*<0.001)). More patients in the PUL cohort required anti-cholinergics and beta-3 agonists post-operatively (8.5% vs 19%, *p*=0.084). With regards to symptom scores, pre-operative MISI severity scores were significantly higher in the PUL group (median 6 vs 3 (*p* = 0.011)) and no other differences were noted.

**Conclusions:**

Patients presenting for HoLEP after prior PUL have significantly higher MISI severity scores. This does not translate to differences in post-operative outcomes compared to those who had HoLEP as their first BPH surgery.

## Introduction

Prostatic urethral lift (PUL) gained traction as an option for treating benign prostatic hyperplasia (BPH) after its approval by the U.S. Food and Drug Administration (FDA) in 2013. It is one of the numerous minimally invasive surgical therapies (MISTs) which aim to control the symptoms of BPH, while preserving antegrade ejaculation. While numerous studies have shown that it does not have the longevity of symptom improvement seen with transurethral resection of the prostate (TURP) or holmium laser enucleation of the prostate (HoLEP), it is still viewed as an option for many patients and it made up about 25% of the procedures performed for BPH in 2021 [1].

Initial results from the L.I.F.T. trial with 140 patients noted that PUL had 88% greater improvement of International Prostate Symptom Scores (IPSS) compared to sham at 3 months [2]. That trial sparked debate about the definition of treatment failure and outcome reporting. Dr. McVary and Kaplan in 2020 noted that the 13.6% 5-year re-treatment rate did not take into account patients returning to medications or those that had implants removed and not replaced, which would bring that number up to 33.6% [3].

Multiple studies have evaluated the effectiveness of TURPs and HoLEPs after failed PUL [4–6]. A trend we have seen in our patients undergoing HoLEP after PUL is the prevalence of irritative voiding symptoms. We sought to investigate the effect PUL has on the patients presenting for HoLEP, and if this leads to different post-operative outcomes.

## Methods

This is a single-institution retrospective analysis of our prospectively maintained BPH registry. Every patient who undergoes a HoLEP at our institution is asked whether they would consent to being a part of our registry (IRB: STU00213284). If they consent, they will have their pre-operative, intra-operative and post-operative information saved on REDCap.

HoLEP was performed by two fellowship-trained endourologists between January 2021 and May 2025. The registry was accessed for this study on June 14th 2025. A two/three lobe modified early apical release technique was utilized depending on the patient’s anatomy and the presence of a median lobe. Any PUL implants that were encountered during the procedure within the adenoma were removed, either through the morcellator or with the use of graspers through the morcelloscope. Patients had a 22 French 3-way Foley catheter placed at the end of the procedure and a same-day void trial (SDVT) was performed unless they had contraindications (capsular perforation, urethral injury, lack of social support).

To create the arms for the study, we first determined the eligible population. We excluded patients if they were lacking key information in the registry. The variables chosen were prostate size, demographics (age, BMI) and if they answered their 1-week follow-up telephone call, as that is when they were asked about early voiding symptoms. Once this was performed, we identified the patients who have had prior PUL. Propensity score matching of prostate size and history of urinary retention was used to match the patients who had prior PUL with patients without prior BPH surgery 1:2.

We analyzed baseline demographics, operative details and post-operative outcomes. We also noted pre-operative and post-operative IPSS and Michigan Incontinence Symptom Index (MISI), if available.

Binary variables were analyzed with Chi-Square tests and continuous variables were analyzed with Wilcoxon rank-sum tests. All statistical analyses were performed using R (version 4.4.2, R Foundation for Statistical Computing, Vienna, Austria). A *p*-value of <0.05 was significant.

## Results

**Fig. 1:**
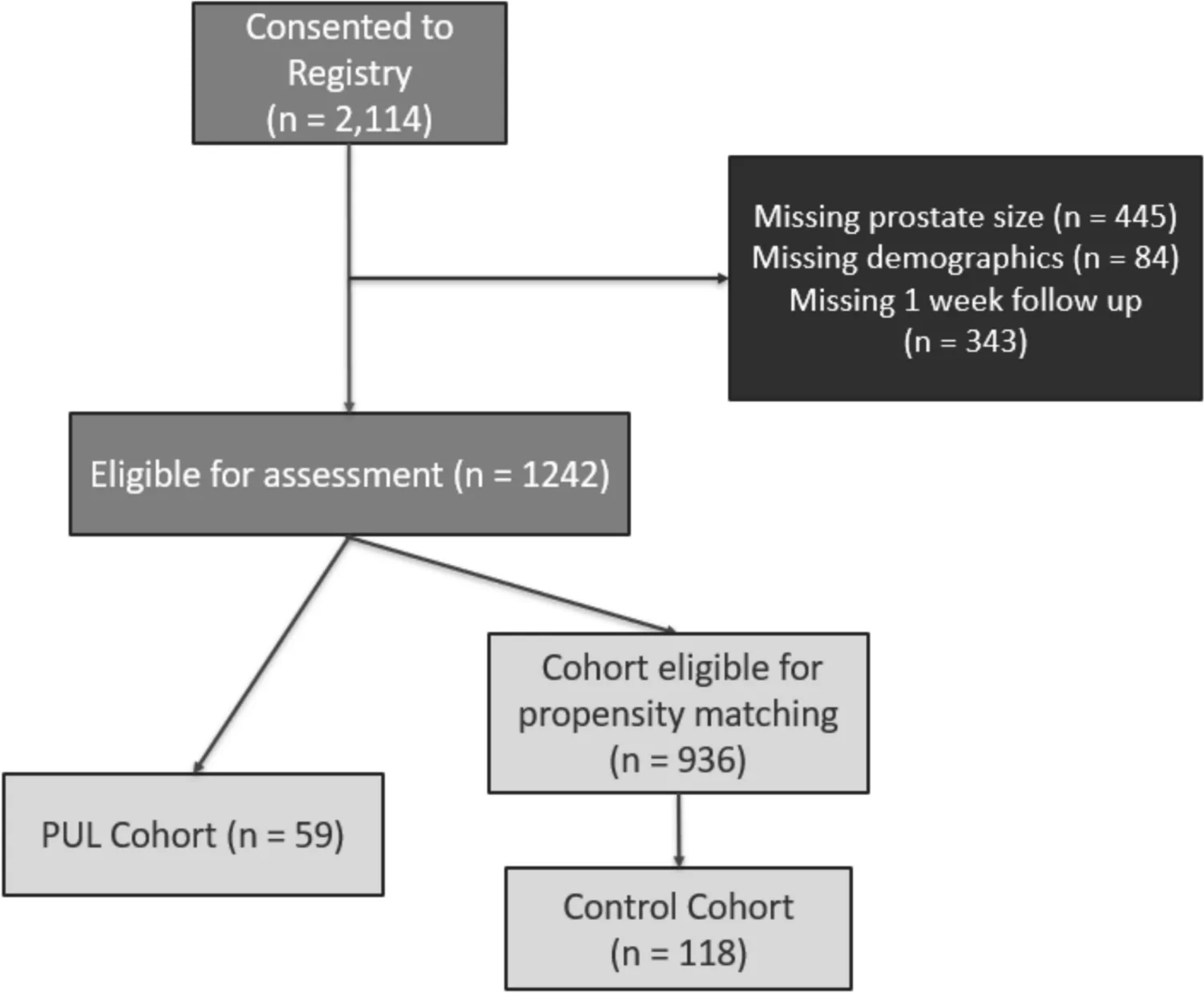
Flowchart demonstrating the patient selection process

**Fig. 2:**
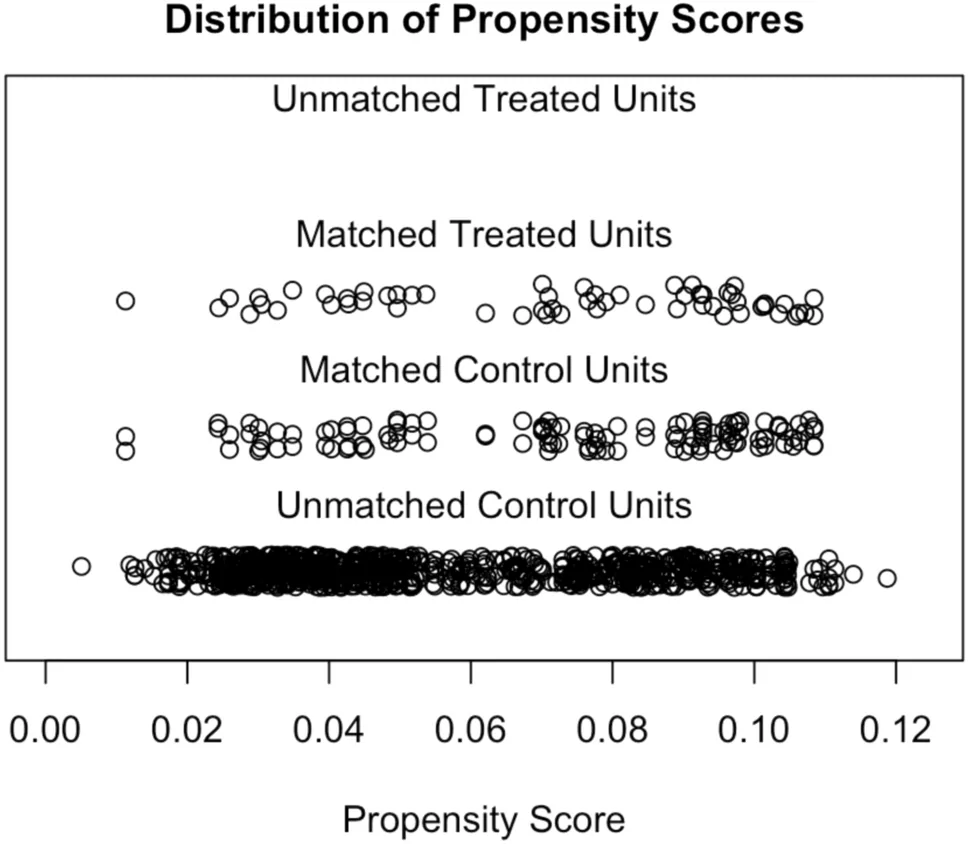
Distribution of propensity scores in the PUL (treated) group and the control group

There were 2114 patients in our registry who underwent a HoLEP between January 2021 and May 2025. After excluding patients with insufficient data for this study, we were left with 1242 patients. There were 59 patients who underwent PUL prior to their HoLEP. They were matched with 118 patients in the control arm. Figure 1 outlined the process and Fig. 2 shows the propensity score for patients in the PUL arm and the control arm.

Baseline demographics are seen in Table 1. The PUL arm had a greater proportion of patients on anticoagulation or antiplatelet medications (27% vs 16%, *p*=0.13), higher American Association of Anesthesia (ASA) scores (*p*=0.036) and fewer patients taking alpha blockers (54% vs 69%, *p*=0.05). The control group was younger (median 70 vs 73 years old, *p*=0.13) and had a lower incidence of urge incontinence (28% vs 40%, *p*=0.258), but these were not statistically significant.

**Table 1:** Baseline patient information in each cohort

	Control *n* = 118	Prior PUL *n* = 59	*p*-value*
Age at surgery^1^	70 (65, 76)	73 (68, 77)	0.130
BMI^2^	28.0 (4.7)	29.1 (5.1)	0.232
History of urinary retention^3^	34 (29%)	18 (31%)	>0.9
Pre-operative indwelling catheter^3^	16 (22%)	5 (13%)	0.4
Pre-operative CIC^3^	5 (7.4%)	7 (21%)	0.1
Pre-operative PSA^2^	4.3 (3.2)	5.1 (4.8)	0.549
History of prostate cancer^3^	3 (2.5%)	3 (5%)	0.402
Prostate size (mL)^1^	81 (55, 135)	80 (57, 135)	0.929
Pre-operative urge incontinence^3^	16 / 57 (28%)	13 / 30 (40%)	0.258
Taking alpha blocker^3^	81 (69%)	32 (54%)	0.050
Taking 5 alpha reductase inhibitor^3^	23 (19%)	13 (22%)	0.157
Taking daily PDE5i^3^	23 (23%)	15 (25%)	0.263
Taking antispasmodic^3^	9 (8%)	5 (9%)	1.000
Taking anticoagulation/antiplatelet^3^	19 (16%)	16 (27%)	0.13
ASA score^3^			0.036*
1 or 2	69 (58%)	22 (38%)	
3 or 4	49 (42%)	37 (62%)	

^1^Median (Q1, Q3)

^2^Mean (SD)

^3^*n* (%)

*p<0.05

Table 2 shows the intra-operative and post-operative outcomes. Operative and enucleation durations were similar between groups. Notably, morcellation took longer in the PUL arm (7 min vs 5 min) and morcellation efficiency was also slower in the PUL arm (7.2 vs 9.8 g/min). Intra-operative complications, rate of passed same-day voiding trials, 90 day post-operative ED visits, and 90 day complications were not different between arms. Patient-reported continence, hematuria and antispasmodic use at 1 week and 3 months did not differ between arms. More patients in the PUL cohort required anti-cholinergics and beta-3 agonists post-operatively (8.5% vs 19%, *p*=0.084). There were similar rates of bladder neck contracture and urethral stricture occurrence.

**Table 2:** Baseline surgery information in each cohort

	Control *n* = 118	Prior PUL *n* = 59	*p*-value*
Operative duration (min)^1^	49 (35, 62)	53 (37, 69)	0.138
Specimen weight (grams)^1^	46 (21, 92)	59 (24, 81)	0.776
Enucleation duration (min)^1^	26 (19, 35)	27 (19, 36)	0.797
Enucleation efficiency (g/min)^1^	1.82 (1.00, 2.73)	1.94 (1.19, 2.68)	0.634
Morcellation duration (min)^1^	5 (2, 9)	7 (3, 13)	0.01*
Morcellation efficiency (g/min)^1^	9.8 (8.1, 12.4)	7.2 (5.1, 10.0)	<0.001*
Received intravesical botulinum^2^	10 (8.5%)	11 (19%)	0.046*
Intra-operative complications^2^	3 (2.6%)	1 (1.7%)	>0.9
Underwent same day void trial^2^	113 (96%)	52 (88%)	0.108
Failed same day void trial^2^	4 / 113 (3.5%)	0 / 52	0.308
No pad usage at 1 week^2^	46 / 111 (41.4%)	28 / 53 (52.8%)	0.869
Dysuria at 1 week^2^	46 / 114 (40.4%)	17 / 55 (30.9%)	0.013*
90-day ED Visit^2^	7 (9.9%)	11 (23%)	0.082
90-day complications^2^	8 (7.8%)	5 (8.8%)	>0.9
90-day Clavien-Dindo grade^2^			>0.9
1	5 (63%)	3 (60%)	
2	3 (38%)	2 (40%)	
90-day anticholinergic/beta-3 agonist prescription^2^	10 (8.5%)	11 (19%)	0.084
Urethral stricture^2^	2 (1.7%)	0 (0%)	0.8
Bladder neck contracture^2^	0 (0%)	1 (1.7%)	0.7

^1^Median (Q1, Q3)

^2^*n* (%)

*p<0.05

**Fig. 3:**
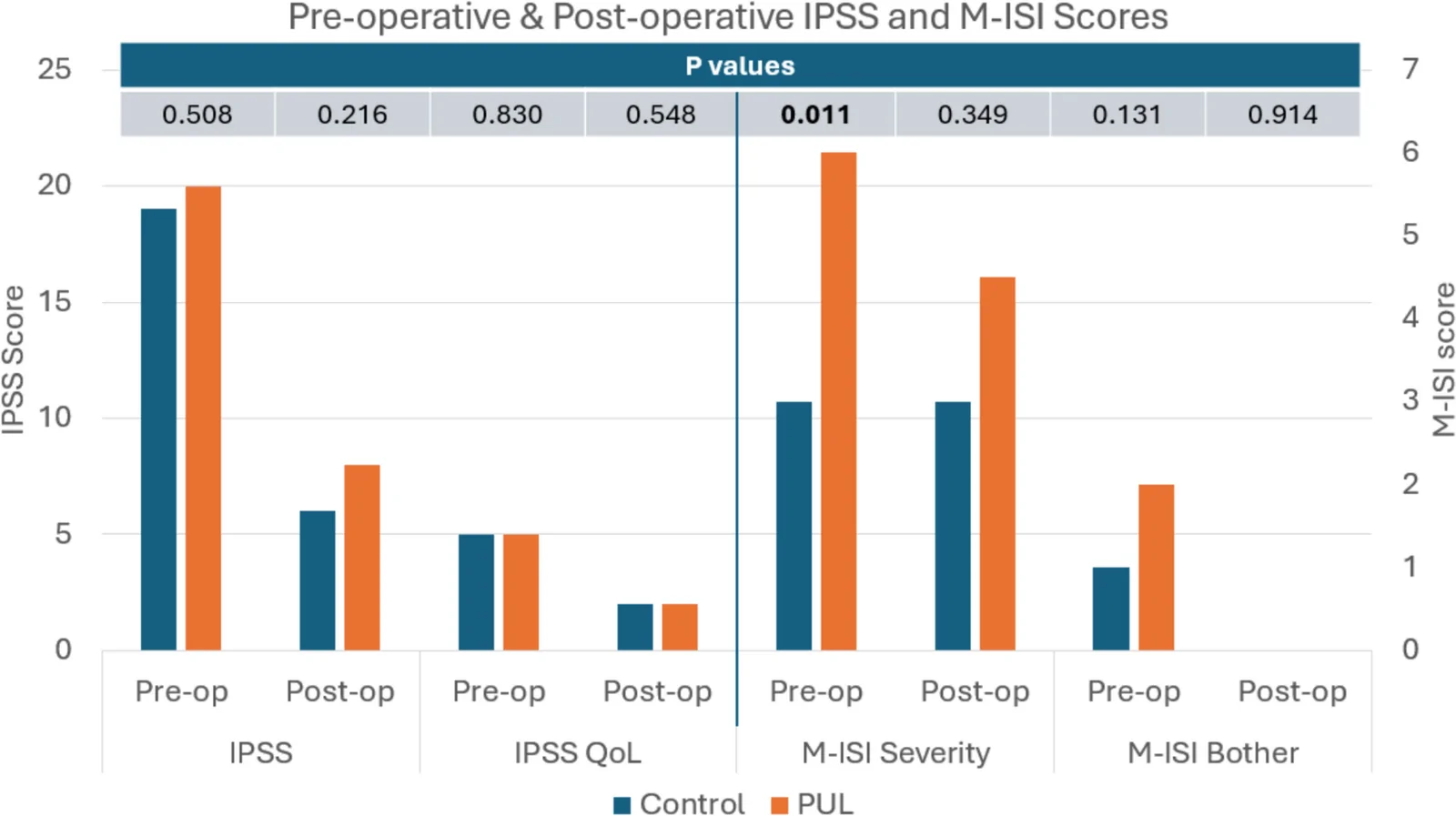
Difference in MISI and IPSS scores pre-operatively and post-operatively in the PUL arm and the control arm

Figure 3 displays the average pre-operative and post-operative voiding symptom scores. Pre-operative M-ISI severity scores were significantly higher in the PUL group (median 6 vs 3 (*p* = 0.011)), and no other differences were noted.

## Discussion

In our study, we found that the mean pre-operative M-ISI scores were greater in the PUL population than in the control cohort. More PUL patients also received intravesical onabotulinum toxinA (OTA), a therapy that we offer to patients with pre-operative urge urinary incontinence. A greater number of patients required post-operative anti-spasmodics, but this did not reach statistical significance.

Intra-operatively, we noted similar operative durations but longer morcellation durations, related to challenges with the PUL implants. This required utilizing the “catch and release” technique employed by Parikh et al. [7] In our experience, this technique minimizes blade malfunction and the need to dislodge PUL implants from within the blade. This did not compromise our post-operative outcomes as patients were able to complete successful SDVT even with prior PUL. There was a trend towards increased 90 day ED visits and anticholinergic prescriptions in the PUL cohort, but these differences were not statistically significant.

It is notable that the PUL group had a greater proportion of patients on anti-coagulation/anti-platelet medications and had higher ASA scores. This suggests that patients may have been offered PUL as a treatment option due to their comorbidities. While patients and some urologists see PUL as a low-risk option to treat BPH, it is important to emphasize that there are notable risks. Significant complications like calcification of implants, re-operations and bleeding have been well reported in the literature, but more gradually developing processes have not been well studied [8, 9]. That being said, we did not note significant differences in intra-operative and post-operative complication rates between groups.

Refractory overactive (OAB) symptoms are a known risk of BPH surgeries, as the obstructing prostate can lead to detrusor overactivity pre-operatively that may be exacerbated during surgical recovery. Our team previously published about the use of OTA at the time of HoLEP in patients with OAB and did see benefit in early voiding symptom scores in the OTA arm [10].

Tsai et al. [11] evaluated the rate of anti-spasmodic prescription after TURP in a population without prior OAB. Their group found that 12% of their 376 patients received at least a 3 month prescription for antimuscarinics or beta-3 agonists within 3 months of their TURP. Older patients and those with intravesical prostatic protrusion were more likely to require pharmacotherapy.

There have been limited studies evaluating post-operative OAB symptoms in PUL patients. Chialastri et al. [12] published one such study retrospectively evaluating post-operative anti-spasmodic prescription in 226 patients after PUL. Compared to pre-operatively, they noted that post-operative anti-cholinergic prescriptions increased from 6 to 8% and beta-3 agonist use increased from 4 to 12%.

With these numbers approaching those of patients who underwent a TURP, it does leave us wondering what is causing this effect. We hypothesize that the presence of foreign body material may be a contributing factor. A study from 1995 evaluating prostatic Urolume Wallstents, showed that 5/34 of the prostatic stents had to be removed due to “unbearable irritative symptoms with poor quality of life” [13]. While PUL implants are not as large as urethral or ureteral stents, the OAB noted in PUL patients may be a manifestation of a similar effect being exerted on the bladder.

There are strengths to this study. All the procedures were performed by a single high-volume HoLEP surgeon that allows for true comparison of the data in both groups, free from variation in surgical technique. The use of propensity score matching allowed the control group to be well balanced with the study arm.

We acknowledge certain weaknesses of the study. Firstly, we are limited by the retrospective nature of the analysis. Despite the prospectively maintained database, we are limited by patient follow-up and despite our cohort sizes, some questions were not answered by some of our patients, and this leads to gaps in the data. Since we did not evaluate the effect on patients who received other therapies for BPH, it is unclear if the effect we are seeing is entirely caused by the presence of the PUL implants or if it is a sequela of prior prostatic surgery. Additionally, there is also a possibility of lead time bias, as patients who underwent PUL may be seen by us later along in their BPH diagnosis compared to those getting a HoLEP upfront, and this may be why their OAB symptoms are more significant. Despite these limitations this study calls to attention an under reported unique symptomatology noted in patients who have undergone PUL with persistent or recurrent LUTS.

## Conclusion

Overall, patients presenting for HoLEP after prior PUL have significantly higher MISI severity scores. Fortunately, we found that higher pre-operative MISI scores in the prior PUL patients do not translate to differences in post-operative outcomes compared to those who had HoLEP as their first surgery for BPH. These findings are beneficial in counseling patients who are considering various treatment options for their symptomatic BPH.

## Data Availability

No datasets were generated or analysed during the current study.
